# Buried K-wires in paediatric fracture fixation: infection and complication rates in a large cohort

**DOI:** 10.1007/s00590-026-04982-w

**Published:** 2026-10-06

**Authors:** Alexa Zimmermann, Johanna Thiele, Christian Prangenberg, Jonas Roos, Davide Cucchi, Sebastian Scheidt, Kristian Welle, Lisa Fiona Roder

**Affiliations:** Department of Orthopedics and Trauma Surgery, University of Bonn, University Hospital Bonn, Bonn, Germany

**Keywords:** Buried K-wires, Paediatric fracture fixation, Kirschner wire infection, Paediatric trauma surgery, Postoperative complications, Infection rate

## Abstract

**Purpose:**

Kirschner wires (K-wires) are widely used for fracture fixation in paediatric trauma surgery. Although infectious complications are uncommon, they remain clinically relevant. Subcutaneous burial of K-wires has been proposed to reduce the risk of infection, but robust data from large paediatric cohorts remain limited. This study aimed to evaluate infectious and non-infectious complications following routine use of buried K-wires in children.

**Methods:**

In this retrospective cohort study, paediatric patients (≤ 18 years) who underwent fracture fixation with routinely buried K-wires at a Level I trauma centre between January 2005 and November 2025 were included. Only patients with complete follow-up, including radiographic assessment and wire removal at the study institution, were analysed. The primary outcome was postoperative infection related to K-wire fixation. Secondary outcomes included non-infectious wound complications, reoperations, and fracture healing.

**Results:**

A total of 634 patients with a mean age of 10.2 ± 4.2 years were included; 96.8% sustained upper-extremity fractures. One superficial infection occurred (0.16%; 95% CI 0.00–0.88%), with no cases of deep infection or osteomyelitis. Secondary skin perforation without infection was observed in 8.36%, and minor wound-healing disturbances in 0.32%. No infection-related reoperations were required, and all fractures healed radiographically.

**Conclusions:**

Routine subcutaneous burial of K-wires in paediatric fracture fixation was associated with an exceptionally low rate of infectious complications and no cases of osteomyelitis in this large retrospective cohort. However, secondary skin perforation occurred in 8.36% of patients, and planned wire removal under anaesthesia represents an additional procedural burden. Given the predominance of upper-extremity fractures and the absence of an exposed-wire control group, these findings should be interpreted within the studied population and do not establish superiority over exposed K-wires.

## Introduction

 Kirschner wires (K-wires) are widely used for fracture fixation in paediatric trauma surgery owing to their versatility, minimally invasive nature, and ease of removal. They are commonly employed for the stabilisation of metaphyseal, epiphyseal, and diaphyseal fractures of both the upper and lower extremities. Despite their favourable biomechanical properties and broad applicability, infectious complications remain a relevant concern following K-wire fixation in children. Reported infection rates after paediatric K-wire fixation vary considerably in the literature, ranging from approximately 1–3% in large contemporary cohorts, with even higher rates reported in selected series [1–7]. Infectious complications encompass a broad clinical spectrum, from superficial pin-tract infections to deep soft tissue infections and osteomyelitis, the latter being a serious complication associated with prolonged treatment, potential reoperations, affection of the growth plate, and long-term morbidity [1]. Recently, Palmetti et al. reported an overall infection rate of 2.4% in a large paediatric cohort treated with exposed K-wires at a tertiary care centre in Italy, highlighting that these infectious complications, although relatively uncommon, remain clinically relevant [8].

However, clinical practice regarding the handling of K-wires varies considerably between institutions and countries, particularly with respect to whether wires are left exposed or buried beneath the skin after fixation. In Germany, subcutaneous burial of K-wires is widely adopted in paediatric trauma surgery. A nationwide survey conducted by the German Society for Trauma Surgery demonstrated that the majority of surgeons prefer buried K-wires for common paediatric fractures, particularly supracondylar humeral and distal radius fractures [9]. More recently, a prospective study comparing buried and exposed K-wires in hand and wrist fixation found no significant difference in infection rates, underlining the ongoing heterogeneity of the literature [10].

Subcutaneous burial of K-wires has been proposed as a strategy to reduce bacterial colonisation at the pin site and limit external contamination. Several studies comparing buried and exposed K-wires have reported conflicting results [10–12]. However, Hargreaves et al. demonstrated lower infection rates with buried wires in a randomised controlled trial [4]. More recently, systematic reviews and meta-analyses have generally suggested a higher risk of superficial infection associated with exposed K-wires [13–15]. Nevertheless, data from large paediatric cohorts treated exclusively with buried K-wires remain limited.

The aim of this study was therefore to evaluate the incidence and characteristics of infectious complications following paediatric fracture fixation using routinely buried K-wires in a large retrospective cohort. By reporting real-world infection rates and clinical outcomes, this study seeks to contribute to the ongoing discussion regarding optimal K-wire management in paediatric trauma surgery.

## Methods

### Study design and setting

This study was designed as a retrospective cohort study. All paediatric fracture cases treated at our institution between January 2005 and November 2025 were screened to identify patients who had undergone K-wire fixation. For eligible patients, clinical and radiological data were retrospectively extracted from the institutional electronic medical records, including operative reports, inpatient and outpatient documentation, follow-up visits, and radiological imaging.

### Ethical considerations

The study was conducted in accordance with the Declaration of Helsinki. It was approved by the local Ethics Committee (ethics approval 406/17). Due to the retrospective design of this study, individual informed consent to participate was not obtained. The study was based exclusively on routinely collected clinical data and involved no study-related intervention or direct patient contact. Retrospective consent from all patients and/or legal guardians was not feasible.

### Study population

**Fig. 1:**
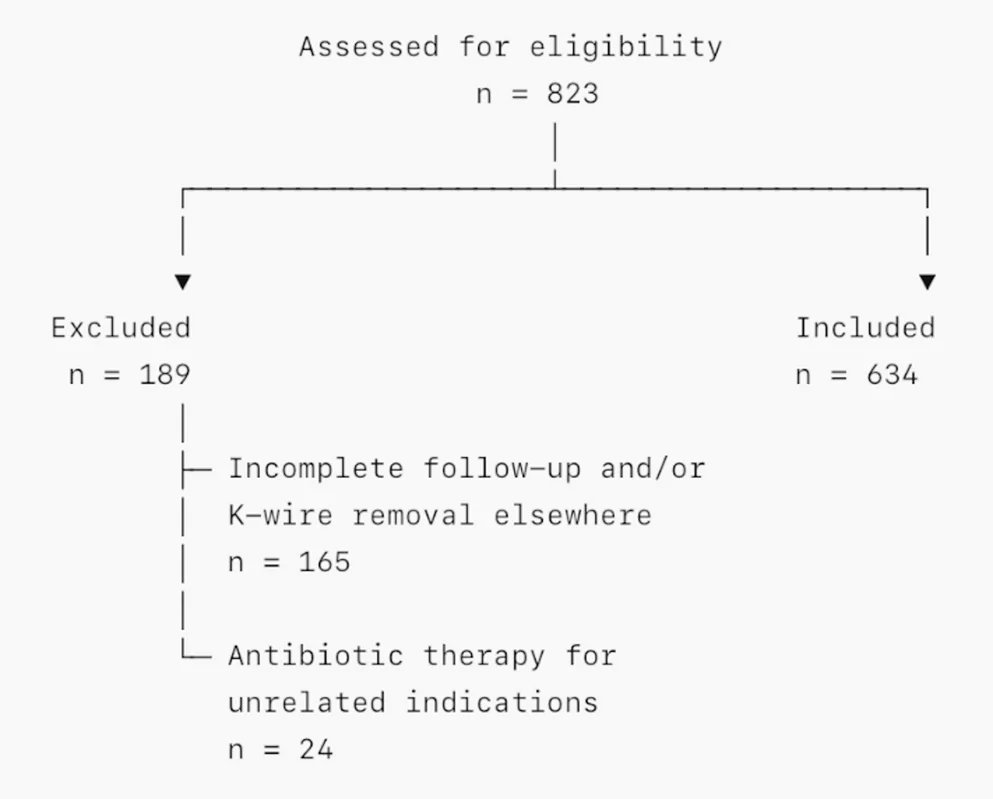
Flow diagram of patient selection. A total of 823 patients were assessed for eligibility. Of these, 189 patients were excluded due to incomplete follow-up and/or K-wire removal elsewhere (*n* = 165) or antibiotic therapy for unrelated indications (*n* = 24). The final study cohort comprised 634 patients

A total of 823 paediatric patients treated with K-wire fixation were identified and assessed for eligibility. After exclusion of 189 patients, primarily because of incomplete follow-up at the study institution and/or K-wire removal performed elsewhere (*n* = 165), and perioperative or postoperative antibiotic therapy for indications unrelated to suspected K-wire infection (*n* = 24), 634 patients were included in the final analysis (Fig. 1). All patients were treated in accordance with institutional protocol, with routine subcutaneous burial of K-wires.

#### Inclusion criteria

- Paediatric patients (≤ 18 years at the time of surgery), irrespective of physeal status or remaining growth potential.
- Traumatic fractures treated with K-wire fixation.
- Implantation and subsequent removal of the K-wires performed at the study institution.
- Postoperative follow-up conducted at the study institution.

#### Exclusion criteria

- Use of additional or alternative fixation methods for the same fracture.
- Patients in whom K-wire removal was performed at another institution or who did not attend follow-up at our centre.
- Patients without X-ray follow-up images.
- Perioperative or postoperative antibiotic therapy administered for indications unrelated to suspected infection at the K-wire site.

### Surgical technique

For distal radius fractures, operative fixation was reserved for fractures with unacceptable alignment, unstable fractures, or fractures in which satisfactory alignment could not be maintained with cast immobilisation alone. Fractures showing acceptable alignment without reduction, as well as fractures with acceptable and stable alignment following closed reduction, were treated non-operatively. Perfect anatomical reduction was not considered mandatory when the residual alignment was considered acceptable for the patient’s age and remaining growth potential.

Surgical treatment was generally performed on an urgent basis, depending on fracture characteristics, soft-tissue conditions, and operating-room availability. The exact interval between admission and surgery was not systematically recorded in this retrospective cohort.

K-wire fixation was performed either percutaneously following closed reduction of the fracture or through an open approach if closed reduction was unsuccessful and open reduction was required. After satisfactory fracture reduction, correct alignment and wire position were confirmed using intraoperative fluoroscopy.

**Fig. 2:**
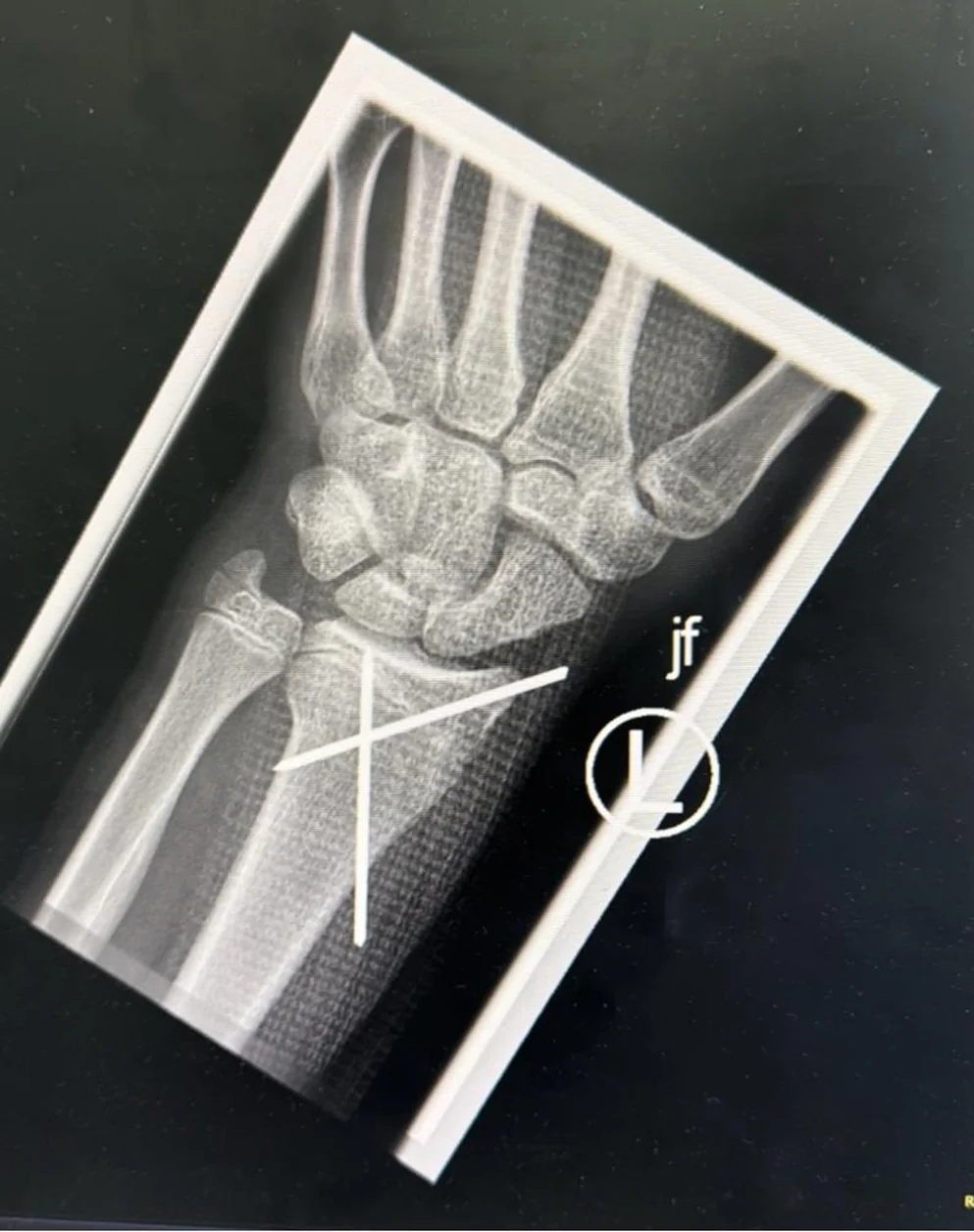
Postoperative radiograph after K-wire fixation. Anteroposterior radiograph of the wrist after distal radius fracture treatment with buried K-wire fixation. The K-wires were placed beneath the skin for temporary stabilization of the fracture fragments. Additional immobilization was provided using a plaster cast

The K-wires were not routinely bent at the bone surface. They were cut with a small intentional overlength to facilitate subsequent removal while keeping the wire ends entirely subcutaneous (Fig. 2). At no point were wires left excessively long or positioned in a way that caused undue tension on the overlying soft tissues. Subsequently, wound closure was performed as required, either by layered closure when necessary or by simple skin closure using sutures or Steri-Strips. A sterile dressing was applied, followed by immobilisation of the affected extremity using a well-padded cast splint.

The institutional practice of routinely burying K-wires remained unchanged throughout the study period.

### Postoperative management and follow-up

In most cases, patients were discharged on the first postoperative day. Standard postoperative wound care instructions were provided, including regular dressing changes performed by the patient’s primary care paediatrician, maintenance of dry wound conditions and avoidance of excessive mechanical stress. Scheduled follow-up visits at our institution were arranged at regular intervals. At scheduled follow-up visits, the cast or splint was removed in most cases to allow clinical inspection of the skin and the area overlying the buried K-wires. Patients and caregivers were instructed to seek earlier clinical review if pain or other local symptoms developed between scheduled visits.

Radiographic follow-up was performed to assess fracture healing. After radiographic confirmation of sufficient osseous consolidation (typically after 6 to 12 weeks), planned removal of the K-wires was planned.

### K-wire removal

K-wire removal at the study institution was performed as an outpatient procedure. According to institutional anaesthesiology practice, removal was routinely carried out under short general anaesthesia. A pneumatic tourniquet was not routinely used. The wires were retrieved through small skin incisions, most commonly using pre-existing surgical scars as entry points to minimise additional scarring.

In many cases, the wire ends were overgrown by soft tissue, which was carefully dissected to expose the wire. If localisation of the wire ends proved challenging, particularly when they were not palpable beneath the skin, additional intraoperative fluoroscopic imaging was used to identify their position. Routine clinical follow-up after K-wire removal was not scheduled in all patients. Patients were instructed to seek prompt clinical review if wound abnormalities, signs of infection, or other postoperative concerns occurred.

### Outcome measures

The primary outcome measure was the occurrence of postoperative infectious complications related to K-wire fixation. Infectious complications were defined according to the criteria used by Palmetti et al. to facilitate direct comparison between cohorts [8] and included superficial soft-tissue infection, deep soft-tissue infection, and osteomyelitis associated with K-wire fixation. Infectious complications were considered present when an infection was clinically documented and required antibiotic treatment and/or surgical intervention. The severity of pin-site infections was assessed using the Checketts–Otterburn classification [16].

Secondary skin perforation was defined as documented postoperative penetration of the overlying skin by a K-wire that had been completely buried beneath the skin at the end of the index procedure. It was diagnosed clinically during postoperative follow-up when a previously completely buried K-wire penetrated the overlying skin, either during scheduled inspection after removal of the cast or splint or during an unscheduled clinical assessment prompted by local symptoms.

Radiographs were routinely obtained as part of fracture follow-up to assess fracture healing and wire position but were not required to establish the diagnosis of skin perforation. In the absence of clinical evidence of infection, these events were classified as non-infectious wire-related complications.

Wound-healing disturbance was defined as a wound-healing disturbance explicitly documented in the medical record following K-wire implantation or removal, in the absence of clinical evidence of infection. Additional secondary outcomes included the need for reoperation and fracture-healing outcomes.

### Statistical analysis

Statistical analysis was primarily descriptive. Categorical variables were summarised as absolute frequencies and percentages. Continuous variables were reported as mean ± standard deviation (SD) or as median [first quartile–third quartile], where available and appropriate. Infection rates and complication rates were calculated as proportions. Exact 95% confidence intervals (CIs) for binomial proportions were computed using the Clopper–Pearson method, given the low number of observed events. No inferential risk-factor analysis was performed for infectious complications because only one infectious event occurred. Exploratory comparisons regarding secondary skin perforation, including age, fracture location, and duration of K-wire implantation, were descriptive.

## Results

A total of 634 patients were included in the final analysis. Of these, 35% were female and 65% were male. The mean age at the time of surgery was 10.2 ± 4.2 years. The vast majority of fractures involved the upper extremity (96.8%). Among upper-extremity fractures, distal radius fractures accounted for 40%, supracondylar humerus fractures for 38%, and other upper-extremity fractures for 22%. Lower-extremity fractures accounted for 3.2% of the cohort and were not further subclassified in the retrospective dataset (Table 1).

Among the 165 patients excluded because of incomplete institutional follow-up and/or K-wire removal elsewhere, the mean age was 11.0 years, 68% were male, and 96% had upper-extremity fractures. These baseline characteristics were broadly comparable to those of the included cohort (mean age 10.2 years, 65% male, 96.8% upper-extremity fractures).

### Infectious complications

Only one postoperative infectious complication was identified (1/634; 0.16%; 95% CI 0.00–0.88%).

This case was classified as a Grade 2 infection according to the Checketts–Otterburn classification and resolved completely after a 12-day course of oral antibiotic therapy after the planned surgical removal of the wires. The patient showed a good clinical response, with complete resolution of symptoms. At no time were clinical, laboratory, or radiological signs suggestive of osteomyelitis observed. No surgical revision was required after the planned removal of the wire, and fracture healing proceeded without delay or sequelae. No cases of deep soft tissue infection or osteomyelitis were detected during the follow-up period (0/634; 0%; exact 95% CI 0.00–0.58% for each outcome).

**Table 1:** Distribution of fracture locations and types in the study cohort

Fracture location/type	Proportion
Upper extremity	96.8% of total cohort
Distal radius fractures	40% of upper-extremity fractures
Supracondylar humerus fractures	38% of upper-extremity fractures
Other upper-extremity fractures	22% of upper-extremity fractures
Lower extremity	3.2% of total cohort

Detailed lower-extremity fracture subtypes and the distinction between percutaneous and open K-wire fixation were not systematically recorded in the retrospective dataset

**Table 2:** Frequency and proportion of infectious and non-infectious outcomes after paediatric fracture fixation with buried K-wires, including exact 95% confidence intervals

Outcome	*n* (%)	95% CI
No infectious or non-infectious local complications	578 (91.17)	88.68–93.26
Secondary skin perforation without infection	53 (8.36)	6.32–10.79
Infectious complication	1 (0.16)	0.0–0.88
Deep soft-tissue infection	0 (0.00)	0.00-0.58
Osteomyelitis	0 (0.00)	0.00–0.58
Wound healing disturbance after planned metal removal without infection	2 (0.32)	0.04–1.13
Infection-related reoperation	0 (0.00)	0.00–0.58

### Secondary skin perforation and wound healing disturbances without infection

In addition to the single infectious complication, secondary skin perforation by buried K-wires was observed in 53/634 patients (8.36%; 95% CI 6.32–10.79%) (Table 2). Secondary skin perforation was first documented at a mean of 15 days after K-wire implantation. Patients with secondary skin perforation had a mean age of 7.0 years, and 50/53 patients (94.3%) had upper-extremity fractures. The mean duration of K-wire implantation was 40 days in patients with secondary skin perforation compared with 45 days in patients without perforation. Patients with secondary skin perforation had a mean age of 7.0 years. The anatomical distribution was similar to that of the overall cohort, and the mean duration of K-wire implantation was not longer in patients with perforation. These cases were associated with local soft-tissue irritation but showed no clinical or laboratory evidence of infection.

Management of secondary skin perforation consisted of local antiseptic treatment, including iodine-based ointment. No patient required systemic antibiotic therapy or unplanned surgical intervention because of skin perforation. After radiographic confirmation of sufficient fracture consolidation, K-wire removal was performed as scheduled. The number of additional outpatient visits or dressing changes attributable specifically to skin perforation was not systematically recorded.

Furthermore, two patients experienced minor wound-healing disturbances following planned K-wire removal (2/634; 0.32%; 95% CI 0.04–1.13%). These cases also showed no signs of infection. One case was resolved with conservative local treatment; the other required minor surgical revision, with a good clinical outcome.

### Additional outcomes

No patient required reoperation due to infectious complications (0/634; 0%; exact 95% CI 0.00–0.58%). All fractures achieved radiographic consolidation. No cases of delayed union or non-union related to infection or wound complications were observed.

## Discussion

The main finding of this study is that routine subcutaneous burial of K-wires was associated with an exceptionally low rate of infectious complications in paediatric fracture fixation. In a large cohort spanning two decades and including 634 patients, only one postoperative infection was observed (0.16%), with no cases of deep infection or osteomyelitis. These findings suggest that routine subcutaneous burial of K-wires may be a safe and reliable technique in paediatric trauma surgery.

Our findings compare favourably with those reported by Palmetti et al., who observed an overall infection rate of 2.4% in a large paediatric cohort treated predominantly with exposed K-wires. In that study, pin tract infections accounted for 16 of 33 infections (48.5%), whereas osteomyelitis accounted for 11 of 33 infections (33.3%) [8]. In contrast, the present cohort showed only one superficial soft-tissue infection and no cases of deep infection or osteomyelitis. Although the infection rate observed in the present cohort was lower than that reported in several cohorts using exposed K-wires, differences in study design, patient characteristics, institutional protocols, and follow-up practices preclude direct comparison. Therefore, the present study does not allow conclusions regarding whether burial reduces the risk of infection. The present findings should be interpreted in the context of heterogeneous existing evidence. Several studies comparing buried and exposed K-wires have reported conflicting results [10–12]. However, Hargreaves et al. demonstrated lower infection rates with buried wires in a randomised controlled trial, and recent systematic reviews and meta-analyses have generally suggested a higher risk of superficial infection associated with exposed K-wires [4, 13, 14]. Hidajat et al. reported a lower risk of pin site infection for buried compared with exposed K-wires, while Chen et al. similarly found significantly higher infection rates after exposed K-wire osteosynthesis [14, 15]. An important limitation when comparing studies is the heterogeneous definition of infection and wound-related complications. In the present study, infectious complications were defined in accordance with Palmetti et al. as clinically documented infections requiring antibiotic treatment and/or surgical intervention. Minor local irritation or secondary skin perforation without antibiotic treatment or surgical revision was classified as a non-infectious wire-related event rather than infection. This distinction is clinically relevant, as other studies have used broader clinical definitions of pin-site infection, including local erythema, discharge, tenderness, or wire loosening, while systematic reviews necessarily combine studies with heterogeneous definitions and follow-up protocols [8, 10, 14].

Deep infections, bone and growth plate involvement represent the most severe complications associated with K-wire fixation and are often associated with prolonged antibiotic therapy, hospitalisation, and surgical reintervention [1, 17, 18]. The low infection rate observed in the present cohort may be attributable to several factors. Subcutaneous burial of K-wires avoids a permanent transcutaneous interface and may therefore reduce external contamination and accidental manipulation. However, burial does not prevent mechanical interaction between the wire ends and the overlying soft tissues, as demonstrated by the 8.36% rate of secondary skin perforation observed in our cohort. Adequate bony purchase and stable wire fixation may also have contributed by limiting wire micromotion and secondary soft-tissue irritation; however, wire purchase was not specifically quantified in the present retrospective study. While the absence of a concurrent control group precludes causal inference, the low infection rate observed in this cohort demonstrates that routine burial of K-wires, within the described institutional treatment protocol, was associated with a favourable infectious safety profile. The relative contribution of wire burial, wire stability, surgical technique, and postoperative follow-up cannot be determined from the present study. Another relevant consideration is the need for a planned secondary procedure to remove the wire. In contrast to exposed K-wires, which can often be removed without anaesthesia, buried K-wires require a secondary procedure for removal [11, 12]. At our institution, this procedure was routinely performed under short general anaesthesia in accordance with local anaesthesiology practice, although alternative anaesthetic approaches may be feasible in selected patients. Although this procedure was performed on an outpatient basis and was generally well tolerated, it is inherently more resource-intensive and may be perceived as a disadvantage of the buried-wire technique. This aspect should be taken into account when weighing the benefits and drawbacks of different fixation strategies, particularly in healthcare systems with limited resources or in patients with increased anaesthetic risk.

Secondary skin perforation was the most frequent non-infectious wire-related complication, occurring in 8.36% of patients. Perforation was documented relatively early, at a mean of 15 days after implantation. Patients with perforation had a mean age of 7.0 years, while the proportion of upper-extremity fractures was similar to that of the overall study population. Furthermore, the mean implantation duration was not longer in patients with perforation (40 vs. 45 days). Although perforation did not result in systemic antibiotic treatment or subsequent infectious morbidity in this cohort, its relatively frequent occurrence represents a clinically relevant drawback of buried K-wires. These events required local wound care and clinical assessment and therefore represent an additional burden for patients, caregivers, and healthcare providers. However, the number of additional outpatient visits or dressing changes attributable to skin perforation was not systematically recorded, and the magnitude of this additional burden therefore cannot be quantified from the present dataset.

Paediatric patients differ substantially from adults in bone biology, immune response, soft-tissue characteristics, and postoperative compliance, underscoring the importance of paediatric-specific data. While adult studies comparing buried and exposed K-wires have reported conflicting results [10–12], evidence focusing on children remains limited. The present study contributes to this body of literature by providing long-term, real-world outcome data from a large cohort treated exclusively with buried K-wires at a high-volume tertiary trauma centre.

Several limitations of this study must be acknowledged. Its retrospective design limits control over potential confounding factors. The requirement for complete institutional follow-up represents a potential source of selection bias. Although excluded patients showed broadly similar baseline characteristics with regard to age, sex, and fracture location, differences in unmeasured characteristics or clinical outcomes cannot be excluded.

Furthermore, routine clinical follow-up after K-wire removal was not performed in all patients, as patients were instructed to return if postoperative abnormalities occurred. Consequently, minor post-removal complications that did not prompt reassessment at our institution may have been underreported.

The long study period of approximately 20 years represents an additional limitation. Although the institutional practice of routinely burying K-wires remained unchanged, individual aspects of surgical technique, perioperative management, and follow-up may have evolved over time. These changes could not be fully reconstructed retrospectively, and a potential influence on complication rates cannot be excluded.

The absence of a concurrent control group treated with exposed wires precludes direct comparison between techniques and does not allow conclusions regarding the superiority of buried K-wires. Furthermore, although definitions of infection were aligned with those used by Palmetti et al. to facilitate direct comparison between cohorts, differences in institutional protocols and follow-up practices must be considered when interpreting differences in infection rates [8]. Another limitation is that K-wire removal in this cohort was routinely performed under general anaesthesia according to local institutional practice. Therefore, the present data cannot assess whether alternative anaesthetic strategies, such as sedation or regional anaesthesia, may reduce procedural burden. These aspects may be relevant when weighing the advantages of buried K-wires against the need for a second procedure for wire removal. Nevertheless, the large sample size, extended observation period, and extremely low number of infectious events provide relevant real-world evidence supporting the safety of routine subcutaneous burial of K-wires in paediatric fracture fixation.

Future studies directly comparing buried and exposed K-wires in paediatric populations should not only evaluate infection rates but also incorporate outcomes such as anaesthesia exposure, procedural burden, patient comfort, non-infectious complications, operating room efficiency, and healthcare resource utilisation to guide evidence-based decision-making.

## Conclusion

In this large retrospective cohort, routine subcutaneous burial of K-wires was associated with an exceptionally low rate of infectious complications, with only one superficial infection and no cases of deep infection or osteomyelitis. However, secondary skin perforation occurred in 8.36% of patients, demonstrating that non-infectious wire-related complications are clinically relevant. In addition, buried K-wires required planned removal under short general anaesthesia. Given the absence of an exposed-wire control group and the predominance of upper-extremity fractures in this cohort, the findings should not be interpreted as evidence of superiority over exposed K-wires or generalised to all paediatric fracture patterns. Within these limitations, buried K-wires demonstrated a favourable infectious safety profile.

## Data Availability

The data that support the findings of this study contain sensitive patient information and are therefore not publicly available. Data can be made available upon reasonable request to the corresponding author, subject to approval by the relevant ethics committee and adherence to applicable privacy regulations.
